# Correction: Elucidating Poor Decision-Making in a Rat Gambling Task

**DOI:** 10.1371/journal.pone.0101541

**Published:** 2014-06-23

**Authors:** The PLOS ONE Staff

There are several errors in the references within the Materials and Methods, Results, and Discussion sections of the manuscript. The corrected sentences that include updated references are below.

Materials and Methods:

In the first paragraph after Equation 1, the sentence should read: “The reward received after taking action *a* in state *s* is described by a state-action pair value Q(s,a), which gradually comes to reflect the ‘goodness’ of selecting action *a* when in state *s* (Ito M, Doya K, 2011; Maia TV, Frank MJ, 2011; Sutton R, Barto A, 1998).”

In the first sentence after Equation 2, the sentence should read: “…where α is the learning rate parameter, *r_t+1_* is the reward received after choosing action *a*, Q(s_t_,a_t_) is the current estimate of the value of choosing action *a* in state *s* at time *t* (Sutton R, Barto A, 1998).”

In the paragraph after Equation 7, the sentence should read: “We choose to model risk in this form, in contrast to some other methods [25, 26] as the present form requires only one parameter and allows learning to reach larger asymptotic values in risky situations.”

Results:

In the section “Decision-making and risk seeking” below Figure 7, the sentence should read: “Risk seeking was implemented by adding a risk-related reward contribution [24] to the actual rewards (see Methods).”

Discussion:

In the first sentence of the eighth paragraph, the sentence should read: “Building on the expanding literature indicating that behavioral traits such as risk seeking affect learning and the prediction error signal [26], [55], we used a reinforcement learning model of the RGT to investigate the relationship between the traits and the decision making performances.”

In the same paragraph as above, the last sentence should read: “The computational model, based on a TD-learning algorithm [13, 14, 15] was modified to include the behavioral traits of risk seeking, reward sensitivity and behavioral inflexibility.”

In the second to last paragraph in this section, the second sentence should read: “Other formalisms such as win-stay loose-shift, Bayesian models or more elaborate TD models could also be explored [21], [22], [23], [25], [26], [58].”

Please note that the bibliography has now changed, and includes three extra references that are not included in the original manuscript:

Ito M, Doya K (2011) Multiple representations and algorithms for reinforcement learning in the cortico-basal ganglia circuit. Curr Opin Neurobiol 21: 368-373.

Maia TV, Frank MJ (2011) From reinforcement learning models to psychiatric and neurological disorders. Nat Neurosci 14: 154-162.

Sutton R, Barto A (1998) Reinforcement learning: an introduction. Cambridge, MA: MIT Press.
